# Financial protection and equity of access to health services with the free maternal and child health initiative in Lao PDR

**DOI:** 10.1093/heapol/czz077

**Published:** 2019-10-23

**Authors:** Somil Nagpal, Emiko Masaki, Eko Setyo Pambudi, Bart Jacobs

**Affiliations:** 1 The World Bank, Phnom Penh, Cambodia; 2 The World Bank, Vientiane, Lao PDR; 3 The World Bank, Jakarta, Indonesia; 4 Deutsche Gesellschaft fur Internationale Zusammenarbeit (GIZ) GmbH, Phnom Penh, Cambodia; 5 Social Health Protection Network P4H, Phnom Penh, Cambodia

**Keywords:** Universal health coverage, free maternal and child health, equity, ethnic minorities, supply-side readiness, non-financial barriers, financial protection, pro-poor

## Abstract

Though Lao People’s Democratic Republic (Lao PDR) has made considerable progress in improving maternal and child health (MCH), significant disparities exist nationwide, with the poor and geographically isolated ethnic groups having limited access to services. In its pursuit of universal health coverage, the government introduced a Free MCH initiative in 2011, which has recently been subsumed within the new National Health Insurance (NHI) programme. Although this was a major national health financing reform, there have been few evaluations of the extent to which it improved equitable access to MCH services. We analyse surveys that provide information on demand-side and supply-side factors influencing access and utilization of free MCH services, especially for vulnerable groups. This includes two rounds of household surveys (2010 and 2013) in southern Lao PDR involving, respectively 2766 and 2911 women who delivered within 24 months prior to each survey. These data have been analysed according to the socio-economic status, geographic location and ethnicity of women using the MCH services as well as any associated out-of-pocket expenses and structural quality of these services. Two other surveys analysed here focused on human resources for health and structural quality of health facilities. Together, these data point to persistent large inequities in access and financial protection that need to be addressed. Significant differences were found in the utilization of health services by both economic status and ethnicity. Relatively large costs for institutional births were incurred by the poor and did not decline between 2010 and 2013 whereby there was no significant impact on financial protection. The overall benefit incidence of the universal programme was not pro-poor. The inequity was accentuated by issues related to distribution and nature of human resources, supply-side readiness and thus quality of care provided across different geographical areas.

**Key Messages**

- The experience of Lao People's Democratic Republic (Lao PDR) with a universal free maternal and child health (MCH) programme showed some progress but remained inadequate on the financial protection and equity fronts, and poor women delivering under the scheme still incurred out-of-pocket expenses, which was a major barrier to utilization.
- The pursuit of universal health coverage by Lao PDR will need focused and specific attention on equity.
- Pro-poor design of a financing programme for MCH services requires due consideration of both supply- and demand-side barriers that impede access for poor and vulnerable population groups, especially those of ethnic minorities. Services most required by these population groups will need to be prioritized, adequately provided and made available when avoiding ‘elite capture’.
- Demand-side efforts could include efforts at addressing access barriers, including providing adequate information, building trust in the facility, reimbursing travel costs and mitigating the opportunity costs of time spent to seek care.

## Introduction

The Lao People’s Democratic Republic (Lao PDR) is a landlocked lower-middle-income country bordering Thailand, Cambodia, Myanmar, Vietnam and China. Its total population is around 6.5 million people with nearly 60% living in rural areas. Lao PDR is an ethnically diverse country, including 49 ethnic groups, of whom the majority live in the rural and remote mountainous areas, with limited communications, transport and social service provision (Clarke, 2001). The ethnolinguistic fractionalization index for Lao PDR, which is the probability that ‘two randomly selected individuals from a population will belong to two different groups’, is high at 0.514 (Alesina *et al.*, 2003). During the period 2005–16, Lao PDR had one of the fastest growing economies in the world—with an average gross domestic product (GDP) growth rate of 7.8% per annum (Masaki *et al*., 2017). Strong economic growth has been accompanied by a significant decline in poverty rates—from 33.8% in 2002 to 22.7% in 2012—but poverty remains concentrated among minority ethnic groups (Pimhidzai *et al.*, 2014).

Lao PDR made consistent and substantial progress regarding key population health outcomes. Life expectancy at birth increased from 49 years in 1980 to 66 years in 2015, whereas infant mortality decreased from 135 per 1000 live births to 50 per 1000 live births during the same period (Lao Statistic Bureau, 2015). The maternal mortality ratio reduced from 546 per 100 000 live births in 2000 to 197 in 2015 (Lao Statistic Bureau, 2015). Despite the measurable improvement, there are high socio-economic and geographic disparities in coverage of health services as well as health outcomes; inequalities relating to wealth and ethnicity are especially pronounced. For example, in 2012, 59% of women from Lao-Tai-headed households had deliveries assisted by skilled attendants, compared with 21% for Mon-Khmer, 18% for Hmong-Mien and 18% for Chinese-Tibetan-headed households (World Bank, 2016). Stunting prevalence and the immunization coverage similarly vary by ethnolinguistic group (Lao Statistic Bureau, 2015).

To achieve the health targets in the Sustainable Development Goals and meet new and emerging challenges, the Government of Lao PDR (GoLP) accelerated its efforts towards universal health coverage (UHC). This included universal access to essential services through development of the Reproductive, Maternal, Newborn and Child Health (RMNCH) service package and the nationwide scale-up of free MCH services during 2013–15 following an initial phase in a limited geographical area. Building on the RMNCH service package, the GoLP is currently implementing the second phase of reforms that aims to ensure that health services of reasonably good quality are accessible to the majority of the population. Under the free MCH initiative user fees by pregnant women or for children under-five were replaced with case-based payments that are financially supported by the government or donors. In addition, poor patients receive provisions for transport and other incidental costs (Prime Minister’s Office, 2012; Prime Minister’s Office, 2014; Ministry of Health, 2017a).

In 2016–17, Lao PDR consolidated the free MCH services together with three other social health protection schemes into a single National Health Insurance (NHI) scheme. The consolidation was carefully designed to ensure uniformity, efficiency, improved management and better risk-pooling. The NHI was quickly rolled out in 15 provinces by the end of 2017, and covered the entire country except Vientiane capital by the end of 2018. However, effective coverage requires a greater focus on equity in service coverage and the smooth integration of the multiple schemes forming the NHI scheme.

This consolidated programme needs to be duly informed by evidence from past implementation of individual schemes. The objectives of this article are therefore to detail the effects of the free MCH programme on equity to inform the consolidated programme. We do so by providing a detailed analysis of the effects of the free MCH programme, with a focus on health centres. Special attention is paid to out-of-pocket (OOP) expenditures for ‘free MCH services’, using a before–after analytical design with difference-in-difference (DiD) method to assess the degree of financial risk protection accorded by the free MCH programme. This is complemented by an assessment of supply-side readiness in terms of human resources and infrastructure of health centres in poor areas populated by ethnic minorities to determine if known access barriers are sufficiently addressed.

### MCH care seeking by ethnic minorities in Lao PDR

In general, there is little published information on maternal and child care outcomes by ethnic minorities in South East Asia (Thummapol *et al.*, 2018) and this is also true for Lao PDR. More information is available on maternal and child health (MCH) services uptake. Ye *et al.* (2010) found a very low utilization of antenatal services by women in Kham district, where the population is predominantly of ethnic minority background. Similar results for MCH services are found in Vietnam (Teerawichitchainan and Phillips, 2008; Goland *et al.*, 2012; Målqvist *et al.*, 2013; Binder-Finnema *et al.*, 2015; Tran *et al.*, 2016) and India (Karan *et al.*, 2014). The reasons for low uptake of health services by ethnic minority women in Asia include geographical constraints as they tend to reside in remote and sparsely populated areas with restricted availability of services, limited means of transportation, low education level, exclusion of women in decision-making practices, limited health literacy and culturally inappropriate practices and suboptimal interpersonal skills of medical personnel (Sychareun *et al.*, 2013; Thummapol *et al.*, 2018). Other impediments to care seeking include language barriers and lack of indigenous persons at the health facilities (Thummapol *et al.*, 2018).

A major reason for not using health services in general and MCH services in particular in Lao PDR is their cost, especially the uncertainty around knowing them beforehand (Ye *et al.*, 2010; Alvesson *et al.*, 2012, 2013; Boudreaux *et al.*, 2014; Chankham *et al.*, 2017). Wagstaff and Lindelow (2014) found that health shocks were common, especially amongst poor households, often necessitating a cut in consumption of basic necessities. This is not surprising as government expenditure on health is low, accounting for 33.2% of total health expenditure (THE) or 0.9% of GDP in 2016 (Ministry of Health, 2018). OOP spending as share of THE remains the single largest source of financing for health, at around 45%, and has been in the same range for the past 4 years (Masaki *et al*., 2017). Health facilities rely on revolving drug fund arrangements for income generation whereby each medicine, laboratory test or medical item is separately billed with a 15% top up on the purchase price. The associated absence of standardized fees creates major uncertainties and affects timely care seeking (Alvesson *et al.*, 2012).

The low government spending on health also impairs effective and efficient delivery of quality health services because of inadequate supplies and equipment, insufficient ability to perform managerial tasks such as supervision and provision of technical guidance and inability to ensure availability of sufficient staff with the required skills (Sychareun *et al.*, 2013). Midwives especially are in short supply and those available tend to have limited basic lifesaving skills (Kanchanachitra *et al.*, 2011) though their availability has considerably improved since 2016 (World Bank, 2019). The rate of qualified health personnel (doctors, nurses and midwifes) is below the international benchmark of 23 professionally trained staff per 10 000 population and this shortage is further intensified by a maldistribution of health workers across provinces and by level and type of health facilities. Currently, about one-third of the doctors, nurses and midwives serve just one-fifth of the population (World Bank, 2016). Despite this shortage and maldistribution, many health workers remain underutilized and see less than one patient a day (Kanchanachitra *et al.*, 2011).

## Methodology

### Data sources

This study analyses data from three secondary data sources, which are, (1) the Community Nutrition Project (CNP) Survey conducted in 2010 (baseline) and 2013 (endline); (2) the Umbrella Facility for Gender Equality (UFGE) Survey from 2014 and (3) the Lao Expenditure and Consumption Surveys 2007/08 (LECS-4) and 2012/13 (LECS-5).

#### The CNP survey

During the baseline (2010) and endline (2013) surveys, information was collected at the levels of health centre, village and household. These surveys were designed to evaluate the impact of the free MCH interventions implemented within the catchment area of 62 selected health centres in six central and southern provinces: Borikhamxay, Khammuane, Savannakhet, Saravane, Champasack and Attapeu. These are high-priority provinces with significant number of poor communities compared with other provinces in Lao PDR. Of the 62 intervention health centres, 20 were randomly selected for inclusion in the survey. These health centres were matched with 20 control health centres in the same provinces, based on a number of criteria including size and catchment area terrain, the ethnic composition and staffing of the HC, and distance from major towns.

For the village survey, five villages (from a panel of all villages in the catchment area of each of the 40 health centres) were surveyed. If there were fewer than five villages, all villages were surveyed. In each of the five villages, the village chief was interviewed using the village questionnaire. This questionnaire provided important information on village characteristics, including distance to health facilities.

For the household survey, only households with at least one child under the age of two and having some information on expenditure on the most recent birth were sampled. The households were sampled from the villages selected for the village survey. In each village, survey teams worked with village chiefs to generate a panel of eligible households—i.e. those with at least one child under age two. From this panel, 15 households were surveyed using interval selection (systematic random sampling with a random starting point). If there were fewer than 15 eligible households, all households were surveyed. Survey teams attempted to survey a target of 75 households per HC catchment area (5 villages × 15 households). If 70 or fewer households were surveyed, an additional sixth village was randomly selected and surveyed as per the methodology of the initial five villages. Ethics approval for the study was obtained from the National Ethics Committee for Health Research, Lao PDR.

During the implementation of CNP, the villages of two health centres (Nongboua and Sob One), each belonging to the intervention and comparison area, received relocation benefits and compensation due to the Nam Theun 2 Hydropower Project implementation. Information concerning these two facilities and associated villages and households was therefore omitted from the analysis. As a result, the baseline survey data included 2766 households from 193 villages for 19 health centres whereas the endline survey data included 2911 households from 191 villages for 19 health centres. Of these 5677 household interviews, 5268 were retained for analysis as 409 were incomplete.

For 40 health centres serving the 207 villages in the original sample, a facility assessment was conducted during May to July 2013. This included questions on the availability of basic amenities, guidelines and staff training, equipment, medicine and facility infrastructure, which was matched to indicators on the WHO Service Availability and Readiness Assessment (SARA) (World Health Organization, 2015). The questions from this survey did not exhaustively cover all indicators from the WHO SARA indicators but were restricted to MCH services. The information collected was used to compile availability and readiness scores as explained in the data analysis section.

#### UFGE survey

This survey was conducted in 2014, led by World Health Organization in collaboration with Ministry of Health and The World Bank. The aim was to capture the service availability and readiness of health facilities, both at primary care and secondary care level, for delivering gender-appropriate health services. In this article, the analysis of UFGE survey only focuses on healthcare workers at the health centre, more specifically on the ethnicity, linguistic skills and gender of the health workers.

The design of the survey was cross sectional. Eighty health centres were randomly sampled with equal probability of selection from a register of all health centres in Lao PDR, and data collection was performed from May to June 2014. This sample was combined with the 40 selected health centres in the CNP survey. There were no significant differences in relevant parameters between the two samples. For each health centre, two health workers (all types and cadres) were randomly sampled without replacement, resulting in a total health worker sample of 232 (eight health workers who were officially on the roster could not be located after three attempts or did not consent for an interview).

The healthcare worker data included modules on ethnicity, language skills, gender, tasks performed at the health centre, demographics, in-service training, hours worked, income (including dual-practice), satisfaction and clinical knowledge, as assessed by vignettes of hypothetical cases related to child health (preventive and curative), maternal health and nutrition.

#### Lao Expenditure and Consumption Survey

The Lao Statistics Bureau has conducted the LECS at 5-year intervals since 1992/93. The purpose of these surveys is to estimate expenditure and consumption of households. The LECS is a nationally representative survey which comprises around 8000 households, stratified by province and village type (urban, rural with road and rural without road). The survey covers all provinces in Lao PDR. The sample is selected using a two-stage sampling process. In the first stage, villages are randomly selected with probability in proportion to their population size. This first stage selection was undertaken prior to the implementation of the LECS 3 survey in 2002/03 when 540 villages were selected. These villages were subsequently revisited in LECS 4 (implemented in 2007/08) and then LECS 5 (implemented in 2012/13). Some of the original 540 villages were merged as part of the Government’s village consolidation programme, and as a result, the LECS 5 survey had 515 villages, whereas the LECS 4 survey had 518. The second stage of sampling involved the selection of 16 households for each of the selected villages.

### Data output measures

#### Maternal health out-of-pocket expenditure data

Information was gathered from the CNP survey on user fees and transportation expenditure associated with antenatal care (ANC), deliveries (including caesarean sections) and postnatal care (PNC). Information for maternal health out-of-pocket (MH OOP) expenditure was derived from six survey questions that were asked to the mother of the youngest child aged under 2 years in the household.

User fees included expenses related to:

- ANC consultations (most recent visit): money paid for (1) blood pressure measurement, (2) urine test, (3) blood test, (4) HIV/STD test, (5) iron tablets, (6) folate tablets, (7) deworming tablets, (8) malaria drugs, (9) bed nets, (10) service charge, (11) unofficial tips and (12) others.
- Deliveries: services received and paid for: (1) blood transfusion, (2) injections for mother, (3) vaccines for baby, (4) medicines, (5) room, (6) meals, (7) service charge, (8) unofficial tips and (9) others.
- PNC consultation (most recent visit): amounts charged for: (1) iron supplementation, (2) folic acid supplementation, (3) vitamin A tablets, (4) child vaccination, (5) curative care, (6) counselling, (7) others and (8) service charge.

Transportation expenditures related to cost for transport to obtain:

- ANC consultation (most recent visit)
- Delivery
- PNC consultation (most recent visit)

#### Household consumption expenditure

Household expenditure data were obtained from the LECS-4 and LECS-5. Using common housing characteristics (building materials and household size) and a common asset list as independent variables, and total average monthly household expenditure as an outcome variable for the LECS4, regression analysis was used to predict total average monthly household expenditure in 2010 (baseline). The same approach was used to predict monthly expenditure in the 2013 (endline) survey using LECS-5 data. The household economic status (quintiles) was derived from the reported per capita household expenditure.

#### Maternal health catastrophic incidence

Maternal health catastrophic incidence is a measure indicating that the household had expenditure for maternal healthcare services (ANC, delivery and PNC) exceeding 10% and 25% of the monthly total household expenditure (Wagstaff *et al.*, 2017).

#### Institutional delivery

An institutional delivery is defined as a delivery at the health facility, namely health centre, district hospital or provincial hospital.

#### Supply-side readiness index

The Availability and Readiness Score of health facilities was calculated as the proportion of available indicators among all the minimum indicators required. For example, a score of 0.5 (50%) implies that the concerned health centre had only half of the stipulated minimum indicators. In the Lao public health system, health centres are categorized into Type A and B, with the former being better staffed and equipped and thus supposedly providing more advanced services than Type B health centres.

### Data analysis

#### Difference-in-difference

DiD evaluation method was used in these analyses to identify the plausible causal relationships between output measures and the free MCH programme. For child *i* between 0 and 23 months of age, our basic DiD estimation strategy is described as a linear specification for outcome *Y_gt_* through

$$Y_{\mathrm{igt}}=\alpha_{g}+\theta_{t}+\beta_{1}G+\beta_{2}t+\cdot \beta_{3}G.t+U_{\mathrm{igt}}+\varepsilon_{\mathrm{igt}}$$

where *α_g_* captures group-level time-invariant (not changing over time) ‘fixed effects’; *θ_t_* captures period time-invariant fixed effects; *G* is an indicator variable for intervention (=1) or comparison (=0) areas; *t* is an indicator variable for baseline (=0) or endline (=1) measurements; the *β*s are the regression coefficients to be estimated; *U_igt_* captures individual, household or village-level factors that vary across groups; and *ε_igt_* captures random error. To control for variables that could potentially confound the relationship between treatment and the outputs of interest, the set of covariates for the calculations consisted of three different levels of individual, household and village. Individual child level characteristics include child’s age and gender. The household level characteristics captured in the regression are mother’s age, mother’s education level, father’s age, father education level, ethnicity, the number of children in the household, total household members and household monthly expenditure. Village-level variables include distance to the nearest road, distance to the nearest health centre and distance to the nearest hospital. For consistency of the analysis, the same set of covariates is applied in analysing various outputs of interest unless otherwise stated.

#### Sample weights

During the analysis, village-level sample weights were used to compensate for the varying probability of inclusion in the survey based on the village population.

## Results

### Household and village surveys

The findings of the household and village surveys are discussed by level of health facility and by economic quintile within each of the baseline and endline survey databases, and also the changes from the baseline to the endline survey. OOP expenditure varied by choice of institutional birth vs non-institutional birth, by level of health facility, and socio-economic status but the degree of variation was large, even within each of these categories (Table 1).

**Table 1:** DiD—average OOP expenditure for maternal health service (in Lao Kip)

Variables	Baseline		Endline		With covariates		
	C	I	C	I	*n*	DiD	SE
Average OOP MH (‘000’ KIP)	98	127	134	233	5268	39	31
Average OOP MH (‘000’ KIP) by place of delivery							
Health centre	259	349	138	203	533	−178	121
District hospital	580	491	475	597	350	183	173
Provincial hospital	1422	1113	2022	1589	139	−2000	1259
Average OOP MH (‘000’ KIP) by economic status							
Poorest	47	47	56	59	1722	−2	24
2nd	47	82	93	146	1312	57	45
3rd	101	72	145	223	1076	115*	58
4th	153	132	262	350	755	17	80
Richest	314	591	361	658	403	−58	194

Data Source: CNP Baseline (2010) and Endline (2013); C, comparison area; I, intervention area; OOP MH, out-of-pocket expenses for maternal health; significance level, *P < 0.05.

The DiD analysis did not find any statistical effect in reducing average OOP expenditure, although there was a reduction in OOP MH expenditure for mothers who delivered at a health centre, both at intervention and control sites (Table 1). Otherwise, there was an increase in average OOP expenditure for maternal health services over the 3-year period, but this was higher in the intervention areas than the comparison area, resulting in a DiD of KIP39 000. The increase in MH OOP of KIP115 000 was significant for mothers of the third quintile residing in the intervention areas.

In line with increasing MH OOP expenditures, the incidence of maternal catastrophic health expenditure at 10% of total household expenditure also grew at intervention sites from 17.4% at baseline to 25.6% at endline or 1.4 percentage points using DiD. Conforming to the programme objectives, it did decrease for women delivering at health centres although this was not significant at 0.8 percentage points (Table 2). Following DiD analysis, there were significant increases in catastrophic MH OOP at the 25% level of total household expenditure, at 3.4 percentage points (Table 3). Although there was a non-statistical reduction in catastrophic expenditure for poorest mothers, women of the second and third quintile were subject to significant increases in catastrophic expenses at the 25% threshold level. The incidence of such expenses at health centres declined by 9.8 percentage points but this was not significant.

**Table 2:** DiD—catastrophic incidence at 10% of monthly household expenditure

Variables	Baseline		Endline		With covariates		
	C (%)	I (%)	C (%)	I (%)	*n*	DiD (pp)	SE
MH catastrophic incidence	13.6	17.4	18.5	25.6	5268	1.4	0.03
MH catastrophic incidence by place of delivery							
Health centre	47.6	52.0	31.9	41.4	532	0.8	0.121
District hospital	84.2	82.9	77.0	85.7	350	20.6	0.119
Provincial hospital	73.2	85.7	100.0	92.2	139	−8.1	0.12
MH catastrophic incidence by economic status							
Poorest	8.9	9.8	9.9	8.0	1721	−3.8	0.029
2nd	6.9	13.1	15.5	22.9	1312	9.8	0.056
3rd	12.9	13.9	20.5	30.6	1077	6.9	0.058
4th	21.4	25.2	34.2	36.9	755	−6.0	0.083
Richest	34.0	45.4	36.2	46.5	403	0.2	0.091

C, comparison area; I, intervention area; pp, percentage points.

**Table 3:** DiD—catastrophic incidence at 25% of monthly household expenditure

Variables	Baseline		Endline		With covariates		
	C (%)	I (%)	C (%)	I (%)	*n*	DiD (pp)	SE
MH catastrophic incidence	6.6	7.8	7.0	13.2	5268	3.4*	0.017
MH catastrophic incidence by place of delivery							
Health centre	20.6	16.2	4.1	11.7	532	−9.8	0.057
District hospital	53.7	47.8	39.2	59.2	350	12.4	0.133
Provincial hospital	64.8	55.2	90.2	78.2	139	−1.2	0.157
MH catastrophic incidence by economic status							
Poorest	4.5	5.3	3.0	4.6	1721	−0.5	0.026
2nd	3.1	9.3	4.9	10.5	1312	7.0*	0.031
3rd	6.0	4.1	7.9	15.9	1077	8.1*	0.041
4th	12.7	7.6	14.3	18.5	755	8.0	0.054
Richest	10.9	22.5	16.7	25.8	403	−0.5	0.085

C, comparison area; I, intervention area; pp, percentage points; significance level *P < 0.05.

Given this significant cost burden for institutional deliveries, it is not surprising that low-economic status was associated with low utilization (Table 4). In the intervention area, in 2013, as many as 81.4% of the births among women of the lowest socio-economic stratum were non-institutional vs 40.1% among women of the richest quintile. In 2013, the institutional births in the intervention sites accounted for 32.7% of all deliveries, up from 18% in 2010. This increase was the most pronounced for health centres where the number of births more than doubled by 2013 (as compared with 2010 baseline). Uptake of institutional deliveries was only significant with DiD for women from the second lowest quintile, at 10.6 percentage points, whereas this was a non-significant 1.1% for the poorest women.

**Table 4:** DiD—institutional deliveries

Variables	Baseline		Endline		With Covariates			
	C (%)	I (%)	C (%)	I (%)	*n*	DiD (pp)		SE
Institutional delivery (any type of health facility)	10.7	18.0	22.8	32.7	5268	0.8		0.04
Institutional delivery by type of facility								
Health centre	2.9	8.1	8.9	17.5	5256	3.4		0.031
District hospital	6.6	5.5	10.7	6.4	5256	−4.2		0.025
Provincial hospital	1.1	4.1	1.8	7.9	5256	1.7		0.011
Institutional delivery by economic status								
Poorest	4.2	8.1	10.0	18.6	1721	1.1		0.029
2nd	6.0	11.2	15.5	27.3	1312	10.6*		0.051
3rd	10.6	12.9	26.4	33.0	1077	−0.8		0.052
4th	18.3	26.3	42.8	43.8	755	−2.8		0.089
Richest	31.0	60.9	60.1	59.9	403	−18.5		0.155

C, comparison area, I, intervention area; pp, percentage points; significance level, *P < 0.05.

Utilization of maternal health services, and in particular institutional delivery, amongst women of the poorest socio-economic quintile, suffers from accessibility barriers and low acceptance due to traditional beliefs and practices (Table 5). Costs are one of the main barriers for not utilizing institutional delivery. However, in addition to financial barriers, non-financial barriers such as convenience of home delivery, time required to reach the facility and distance to the facility were also often cited as reasons for non-institutional births.

**Table 5:** Reasons provided for non-institutional delivery by women of the poorest socio-economic quintile (in %)

Reasons	Baseline			Endline		
	C	I	Total	C	I	Total
Convenience to stay home	46.0	40.5	43.1	41.2	33.2	37.5
Not able to reach facility in time	15.7	17.7	16.8	21.5	30.4	25.6
Traditional practice to deliver at home	18.7	24.4	21.8	14.6	21.1	17.6
No money	11.3	9.6	10.4	10.5	8.1	9.4
Distance to facility	7.3	7.0	7.2	10.8	5.2	8.2
No trust in health providers	0.5	0.1	0.3	0.4	0.3	0.3
Other	0.4	0.8	0.6	1.0	1.8	1.3
No response	0.0	0.0	0.0	0.1	0.0	0.1

Source: CNP Baseline (2010) and Endline (2013); C, comparison area; I, intervention area.

**Figure 1:**
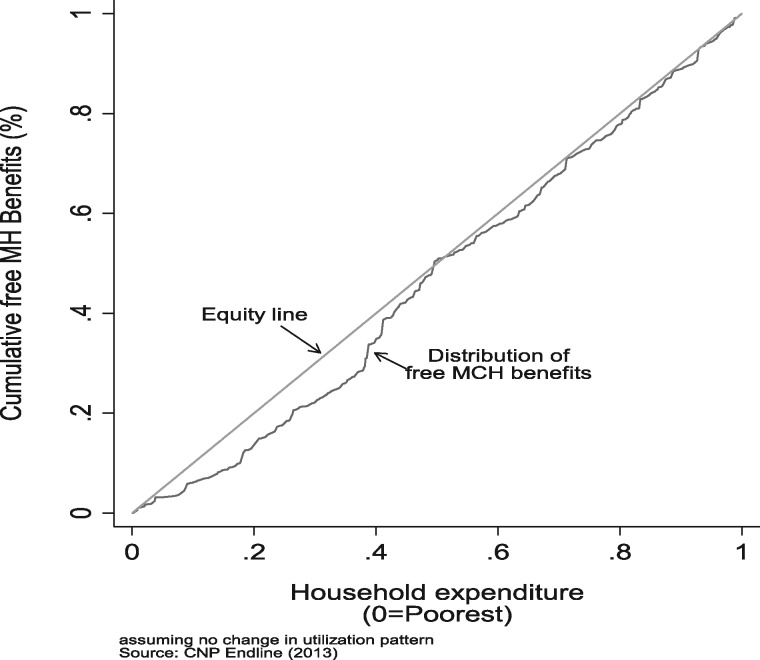
Distribution of free maternal health benefits by household’s socio-economic status (2013).

The results augur not too well in terms of the impact of the free MCH scheme on financial protection, utilization of health centres and reduced OOP expenditure by the poorest households. Figure 1 shows that the universal programme did see a higher benefit utilization among the richer quintiles.

**Figure 2:**
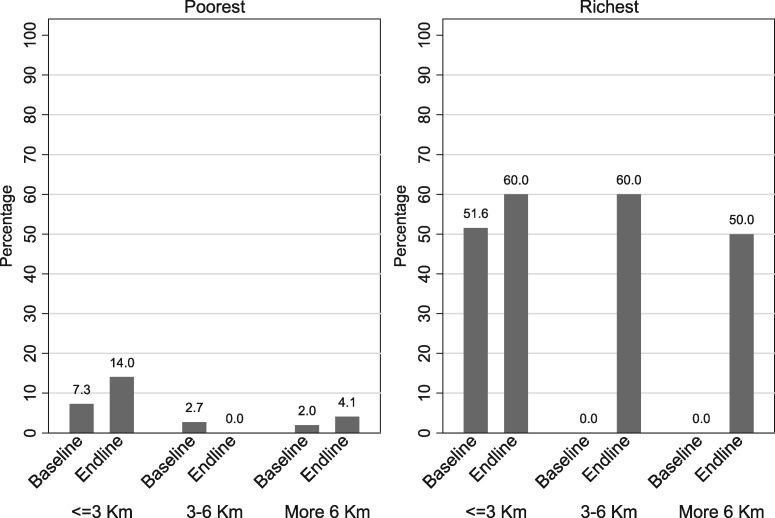
Equity in institutional births by distance from road and economic quintile, 2010 and 2013.

Although utilization of health services increased across the board over the last few years, there are significant differences by place of residence and socio-economic status (Figure 2). The discrepancies are not only caused by differences in the incidence of reported illness, but especially by care seeking behaviour.

### Health facility assessment

Results of the health centre survey revealed that the service readiness to provide MH care is suboptimal. Few health centres had lifesaving maternal health drugs available, such as uterotonic drugs to treat postpartum haemorrhage, injectable antibiotics to treat infections or magnesium sulphate to treat eclampsia or pre-eclampsia (Table 6).

**Table 6:** Service-specific readiness indicators of health centres

MH services	Availability at health centres (%)
Any staff trained in ANC in last 2 years	63.4
Any staff trained in safe motherhood in last 2 years	63.4
Soap and running water or alcohol-based hand rub	63.4
Safe disposal of infectious wastes	68.3
Safe disposal of sharps	34.1
Latex gloves	92.7
Injectable antibiotic	24.4
Injectable uterotonic	22.0
Urine dipstick for protein	17.1
Haemoglobin measurement	12.2
Magnesium sulphate injectable	2.4

**Figure 3:**
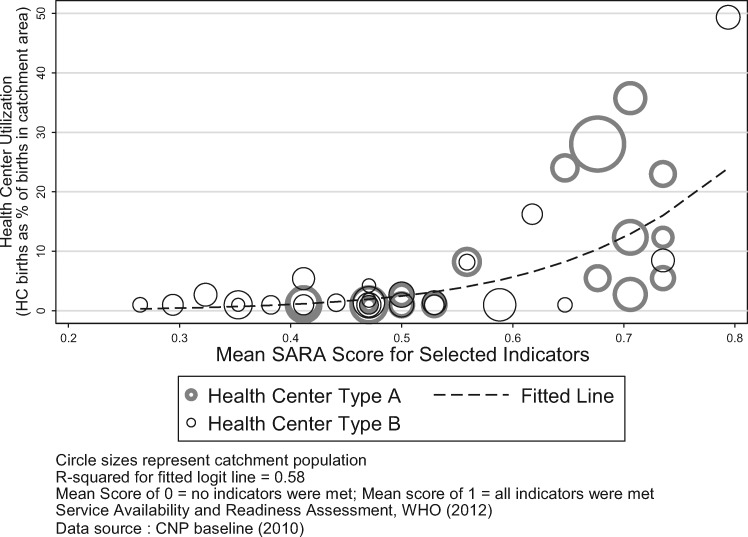
Utilization and service readiness.

Using linear regression to look at the association between supply-side readiness and uptake of maternal healthcare, the survey results indicate that utilization is positively associated with service readiness. There was a substantial uptake in births at health centres with high service readiness compared with health centres with low service readiness (Figure 3).

**Figure 4:**
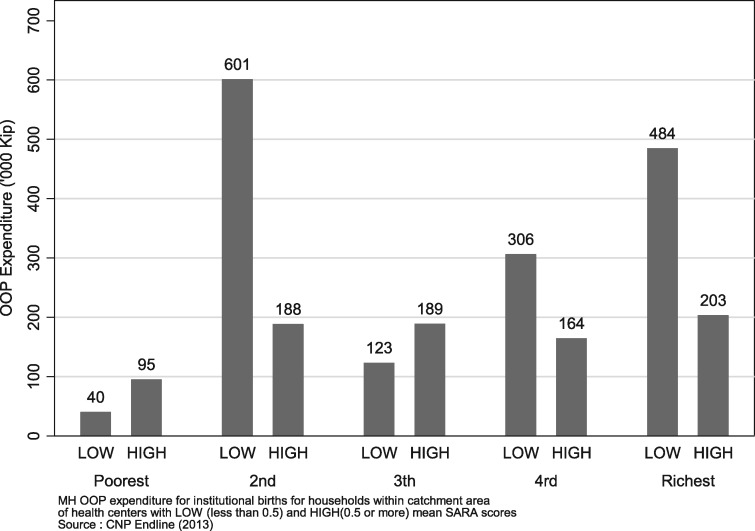
MH OOP expenditure by socio-economic status and health centre readiness.

Households within the catchment areas of health centres with high service readiness scores tended to enjoy better financial protection, measured as lower OOP expenditure for institutional births, compared with households in catchment areas of health centres with low service availability readiness scores (Figure 4). Improved supply-side service readiness was thus not only associated with increased utilization but also improved financial protection.

### Healthcare workforce assessment

The workforce survey found that health centre staff members by and large mirrored the diverse ethnicity of the country—approximately 61% were Lao, 10% were Khmu and 9% were Hmong—which was comparable to the national distribution of these ethnicities at 55%, 11% and 8%, respectively. However, even though the ethnic composition of health centre workers mirrored the national aggregate composition, there was a mismatch in the placements of health workers such that only 56% of health centre workers were of the same ethnicity as the most common ethnicity in the catchment population. Importantly, given that the catchment population is itself multi-ethnic in most cases, a further 29% of the health workers had the same ethnicity as either the second or third most common ethnicity in the catchment population.

Linguistic concordance may be a more important factor as health workers and their patients need to be able to communicate. The survey found that although 85% of staff members were able to speak the most common language of the catchment population, typically the Lao national language, much fewer were able to speak another language—only 31% spoke the second most common language of the catchment population and only 7% were able to speak the third most common language. Fifteen percent of health workers were not able to use their first language to communicate with most of the people in their catchment population.

With regards to gender and employment, this survey found that women made up the majority (57%) and that their aggregate compensation was equal to that of male counterparts, although they were on average 3 years junior to their male counterparts. Certain positions and grades are dominated by women—midwives and MCH providers—but most health centre heads, 64%, were male. Of all the health centre workers who conducted a delivery (either at the facility or at home) within the last 3 months of the survey, 42% were male, typically medical assistants and nurses.

## Discussion

Lao PDR is now gearing up for its next generation of reforms in the health sector in order to address challenges that persist in the sector. This is an opportunity to continue with some proven good practices, whereas also reviewing what can be further improved upon. This article provides some guidance in that direction, using a synthesis of multiple surveys that have not yet been analysed together. The free MCH initiative introduced in 2011 was an important building block for the country’s journey towards UHC. The essence of this initiative was that user fees and other expenses which were hitherto required to be paid OOP by households, for health services required by pregnant women or children, were replaced with case-based payments paid by or through the government or donors.

However, in accordance with similar initiatives elsewhere (Witter *et al.*, 2016; Tama *et al.*, 2018; Dalinjong *et al.*, 2018) medical services were not accorded for free. In Ghana, women were still charged despite fee waivers because of shortages in supply of drug and other consumables or because of using tests not covered under the scheme (Dalinjong *et al.*, 2017, 2018). It is likely that this also happened in our study setting as OOP expenses at health centres with high degree of readiness were much lower than at facilities that had poor readiness scores. On the other hand, reimbursement of providers under the scheme may not have been timely whereby facilities were unable to replenish stocks, as reported from Kenya (Tama *et al.*, 2018). Delays or inadequacy of resources for the Free MCH Programme may have been a driver of the supply-side readiness challenges in Lao PDR, and also for the persistence of high OOP expenses.

Possible reasons for the high variation in OOP spending for institutional deliveries may include clinical differences in complexity of the delivery, as well as varying levels of supply-side readiness and funding of health facilities, whereby the patients’ household had to finance the gap in availability of supplies. Since variation was greatest at provincial hospitals where supply-side readiness was likely to be adequate, it is also possible that add-on services not covered under the free MCH programme were provided, resulting in variable user fees paid by the household.

The increasing degree of variation in OOP expenses for vaginal deliveries by higher level—and thus better equipped—facilities also suggests that the free MCH scheme does not cover sufficient ancillary services, or there may have been expenses on consumables and tests used during covered procedures. Conversely, the absence of standard care guidelines to be followed by health providers for such deliveries is likely to induce the use of unnecessary and more expensive medical tests and drugs. In Nepal, such practices were tempered by use of a birthing centre accreditation framework (Bhatt *et al.*, 2018). Without improved availability of such commodities, improvements in health outcomes of mothers and children in the SDGs may be hard to achieve.

Following the introduction of the Free MCH Programme, there was a reduction in OOP expenses at health centres but this was not significant. Further, contrary to the scheme’s objectives there was an increase in OOP expenses for maternity services. There was also a non-significant decrease in catastrophic expenses at the 10% level for the poorest quintile (by 3.8 percentage points) but this was only 0.5 percentage points for the 25% level. Catastrophic maternal health expenses (at the 25% threshold level) increased significantly overall and especially for women of the second and third socio-economic quintile. Thus the scheme did not achieve financial protection, contrary to findings from elsewhere (Witter *et al.*, 2016). In addition, from an equity perspective, the initiative did not attain its full potential as the better-off secured a disproportionate share of the programme benefits, similar to findings from Nepal (Bhatt *et al.*, 2018). In Ghana results were mixed depending on the criteria used to measure equity impact (Witter *et al.*, 2009).

In accordance with its objectives, the Free MCH Scheme did increase institutional deliveries, as observed elsewhere (Delamou *et al.*, 2015; Witter *et al.*, 2016; Dalinjong *et al.*, 2017; Tama *et al.*, 2018; Bhatt *et al.*, 2018; Gitobu *et al.*, 2018). The proportion of the poorest women delivering at a health facility doubled for those residing within 3 km of the health facility or further than 6 km away but was still only 14% for the former and 4% for the latter. This contrasts with the richest women of whom at least half had an institutional delivery.

Obviously, there is a need to work on challenges facing the health system, not only because of the low uptake of delivery services, despite partial fee waivers, but also because of the very low care seeking in general as indicated by a comparison with Cambodia. Compared with results from a similar survey in Cambodia, the rates of care seeking are much lower in Lao PDR than in Cambodia (Table 7).

**Table 7:** Outpatient utilization of health services in the past 4 weeks, by economic quintile, 2012–13 (in %)

	All	Poorest	Q2	Q3	Q4	Richest
Lao PDR						
Any illness or injury	10.0	9.2	9.5	9.1	10.5	11.6
Seeking care when ill	30.9	23.6	27.0	29.4	32.9	39.1
Seeking care in public facilities when ill	24.7	20.0	22.9	24.7	27.2	27.6
Seeking care in private facilities when ill	10.2	5.2	8.4	8.0	10.0	17.5
Cambodia						
Any illness or injury	14.5	13.8	13.4	14.9	15.2	15.1
Seeking care when ill	86.1	83.0	81.2	85.3	87.6	92.2

*Sources*: World Bank calculations, based on Lao Expenditure and Consumption Survey (LECS) V 2012/13, Cambodia Socioeconomic Survey (2014).

Although not mentioned by the interviewees in our surveys, there may have been a low perception of quality of care, which was identified by other studies in Lao PDR as a major determinant for not seeking care (Alvesson *et al.*, 2012; Sychareun *et al.*, 2013). The considerably higher occurrence of institutional deliveries at facilities that were better equipped reinforces this line of thinking. The need to strengthen the supply side to improve the uptake of institutional deliveries—and reduce OOP expenses—has also been recommended by others (Witter *et al.*, 2009; Bhatt *et al.*, 2018; Tama *et al.*, 2018). Such investments include appropriate equipment, staffing, training, essential medicines and commodities. It may also require implementation of standard treatment guidelines and introducing licensing and accreditation systems. These improvements could reduce demand-side barriers related to physical access, ethnolinguistic and gender issues, and a reliance on OOP payments, which currently exacerbate inequalities in health service utilization, and result in inequity in access to essential maternal and health services.

The ethnic and linguistic diversity of Lao PDR, especially in rural areas, also presents an inherent challenge for frontline healthcare service delivery. The ethnolinguistic mismatch observed in our study may hamper optimal use of health services (Binder-Finnema *et al.*, 2015; McKinn *et al.*, 2017). Targeted recruitment of health workers from rural ethnolinguistic groups, coupled with an intentional deployment back to their communities, may mitigate this challenge, and has been used successfully in other countries such as Vietnam (Vujicic *et al.*, 2010). The high share of male health workers involved in providing maternity services (at 42%) is also unusual given that in the overall health system, most health workers are female (especially among those who are midwives and/or have MCH responsibilities). The low utilization of maternity services may be compounded by such missed opportunities for greater gender sensitivity and needs better understanding of sociocultural practices. Considering that the HC workforce already includes sufficient numbers of female health workers trained in MCH services, it may be helpful to augment their skills and to have sufficient numbers of female birth attendants available in health facilities at all times, where feasible, to allow pregnant women a greater chance to be attended by a female birth attendant.

Health insurance can, of course, play an important role in incentivizing facilities to improve supply-side readiness so that these reforms are linked, but due attention would need to be paid in the overall reform plan and a sequence of the reforms in order not to miss out on the supply-side’s capacity, readiness and even motivation to deliver these services that are more oriented towards the poor and vulnerable groups.

### 

#### Research limitations

As with all researches, some questions are answered whereas others emerge. For the case of the Free MCH Scheme in particular and operations of the Lao health system in general, a series of research issues would benefit from further exploration. These relate to ensuring a steady and reliable supply of drugs and consumables, means to reduce supplier induced demand at higher level health facilities, ways to facilitate institutional deliveries for remotely living women with consideration of previous interventions (Eckermann and Deodato, 2008), effects of prior knowledge of Free MCH Scheme on uptake of associated services, which financial and non-financial strategies work best to ensure sufficient employment of medical staff with the required profile in remote poor settings, how strategic purchasing may guide best practices concerning supply-side readiness and cost containment, etc. Because traditional practices concerning deliveries appear an important factor for foregoing institutional deliveries, it could be considered to assess whether improved health literacy or incorporation of harmless traditional practices during birthing at health facilities or a combination of both has a desired effect. We also did not control for prior knowledge of the free MCH scheme which could be an important factor affecting utilization, and may be an added dimension of future research in this area.

### Policy implications

Lao PDR’s National Health Insurance Strategy 2017–20 provides a vision and clear directions for the successful roll out of an integrated NHI system, including free MCH services (Ministry of Health, 2017b). As stipulated by the National Health Insurance Law approved in 2019, the NHI Bureau (NHIB) has to fulfil key operational functions in order to provide effective and equitable coverage as well as play its role in stewardship for the health system as a whole (National Health Assembly, 2018). This article has analysed the experience from the free MCH programme, which did achieve some progress and yet remained inadequate on the financial protection and equity fronts. Using multiple surveys, we showed that financial protection was not achieved, that equity did not improve, that supply-side readiness was inadequate in poor settings, but that service uptake correlated with quality of care and that staff members and their activities did not correspond to the gender and ethnolinguistic requirements. This reinforces an important policy issue that free care at the point of delivery alone does not equates UHC and that health system issues require due consideration (Tangcharoensathien *et al.*, 2018). The performance of the Free MCH may necessitate also a closer look at the ability to enable UHC by the other schemes’ that make up the NIH system. However, having looked only at selected elements of the health system a more profound assessment—particularly concerning staffing, financing, drugs and consumables—may be required to enable formulation of sound policies to advance UHC successfully.

What emerges thus far is that to be effective, appropriate implementation capacity of NHIB will be a prerequisite to ensure that the NHI policy is translated into reality on the ground; i.e. ensure affordable access to quality health services, especially for poor people. This requires adequate and sustainable financing of the NHI programme to address supply-side constraints and to reduce rationing and inadequate provision at facility level. Inadequate or delayed funding for health facilities to carry out this mandate could create a disproportionate impact on the poor and underserved groups. In addition to addressing financial and non-financial barriers and making efforts to model the health system more ‘service ready’ for UHC, efforts will also be needed on the demand side to promote uptake of services among the poor and those in remote areas. Foremost there is a need to ensure that OOP expenses for maternal health services are at an affordable level. Even when services are available and affordable (or free), there remain significant access barriers, including adequate information, travel costs and opportunity costs for care seeking. Targeting could be considered for some specific interventions to reduce access barriers for the poor. Evidence-based and pro-poor design of the NHI benefit package is also important. This requires due consideration of population health and primary care services and addressing income and geographic barriers. For ethnic minorities, health facilities should be made more culturally acceptable by posting female staff members from the same ethnolinguistic group.

A role for NHIB to undertake strategic purchasing is critical to improve access and quality of services, especially for the poor and vulnerable groups, as well as regular reviews of the pricing and case-definition of services—as there may be mismatches and inappropriate incentives in the actual costs of maternal health services and reimbursement schedules. Finally, strong monitoring systems are required in order to track coverage of essential services, assess the degree of financial risk protection especially among the poor and vulnerable, to understand the causes of catastrophic and impoverishing health expenses, and to inform the government on what incremental improvements would be helpful. This should signal any inequitable aspects of the programme performance to correct them in time.

## Conclusion

Lao PDR is at a critical juncture of health financing reforms in its path to UHC. In order to successfully transform the current health system to achieve UHC, it is imperative to implement the NHI system in conjunction with the supply-side financing needs to ensure equitable delivery and financing of essential services and to promote quality and service readiness at all levels of services. Careful design and implementation of the NHI benefit package requires due consideration of a number of issues, including the health and cultural needs of the population and their income, geographic and other barriers so that services are available to the poor and underserved and that ‘elite capture’ is avoided and the benefits of the programme are instead distributed in a pro-poor manner. Due policy attention to the lessons from the country’s own past would help catapult Lao PDR forward on its path to UHC with due financial risk protection, equity and inclusiveness.

*Ethical approval.* The underlying baseline and endline surveys conducted by World Bank’s Community Nutrition Project received ethical clearance from the National Ethics Committee for Health Research, Ministry of Health, Government of Lao PDR.
